# Positive selection and recombination shaped the large genetic differentiation of *Beet black scorch virus* population

**DOI:** 10.1371/journal.pone.0215574

**Published:** 2019-04-25

**Authors:** Shirin Farzadfar, Reza Pourrahim

**Affiliations:** Plant Virus Research Department, Iranian Research Institute of Plant Protection (IRIPP), Agricultural Research, Education and Extension Organization (AREEO), Tehran, Iran; Keele University Faculty of Natural Sciences, UNITED KINGDOM

## Abstract

*Beet black scorch virus* (BBSV) is a species in the *Betanecrovirus* genus, in family *Tombusviridae*. BBSV infection is of considerable importance, causing economic losses to sugar beet (*Beta vulgaris*) field crops worldwide. Phylogenetic analyses using 3′UTR sequences divided most BBSV isolates into two main groups. Group I is composed of Iranian isolates from all Iranian provinces that have been sampled. Chinese, European, one North American and some other Iranian isolates from North-Western Iran are in Group II. The division of Iranian BBSV isolates into two groups suggests numerous independent infection events have occurred in Iran, possibly from isolated sources from unknown host(s) linked through the viral vector *Olpidium*. The between-group diversity was higher than the within-group diversity, indicating the role of a founder effect in the diversification of BBSV isolates. The high *F*_*ST*_ among BBSV populations differentiates BBSV groups. We found no indication of frequent gene flow between populations in Mid-Eurasia, East-Asia and Europe countries. Recombination analysis indicated an intra-recombination event in the Chinese Xinjiang/m81 isolate and an inter-recombination breakpoint in the viral 3′UTR of Iranian isolates in subgroup IranA in Group I. The *ω* ratios (*dNS*/*dS*) were used for detecting positive selection at individual codon sites. Amino acid sequences were conserved with *ω* from 0.040 to 0.229 in various proteins. In addition, a small fraction of amino acids in proteins RT-ORF1 (p82), ORF4 (p7b) and ORF6 (p24) are positively selected with *ω* > 1. This analysis could increase the understanding of protein structure and function and *Betanecrovirus* epidemiology. The recombination analysis shows that genomic exchanges are associated with the emergence of new BBSV strains. Such recombinational exchange analysis may provide new information about the evolution of *Betanecrovirus* diversity.

## Introduction

Soilborne viruses, especially those persistently transmitted by plasmodiophorid and chytrid vectors, are economically important and cause considerable losses to sugar beet (*Beta vulgaris*) production worldwide [1]. *Beet black scorch virus* (BBSV) belonging to the genus *Betanecrovirus*, family *Tombusviridae* [2, 3, 4], was first reported in Chinses Inner Mongolia [5, 6, 7]. This virus induces severe systemic disease symptoms of black scorching leaves tips, necrotic fibrous roots and severe stunting of affected sugar beet plants in fields throughout North-West and Eastern China. Later, BBSV was reported from the USA [7], and Europe [1] with no black scorching on the leaves but showing exacerbated symptoms similar to that of rhizomania as induced by *Beet necrotic yellow vein virus*-BNYVV (genus *Benyvirus*, *Benyviridae* family) [1, 7]. Symptomless infections have also been observed in sugar beet [7]. BBSV is transmitted by the chytrid vector *Olpidium brassicae* zoospores in a non-persistent manner [8]. In addition, the virus is also mechanically transmissible to a number of herbaceous hosts and induces necrotic local lesions on *Chenopodium spp*., *Tetragonia expansa* and *Spinacia oleracea*. *Nicotiana spp*., *Solanum lycopersicum*, *Physalis floridana* and *Lactuca sativa* are asymptomatic hosts [9].

BBSV has a polyhedral particle, 28 nm in diameters which encapsidates a positive-sense, single-stranded (ss) genomic (g) RNA consists of 3644 nucleotides. The genome lacks a 5′ cap structure and a 3′ poly A tail. BBSV contains six open reading frames (ORFs) [3]. ORF1 encodes a 23 kDa protein (p23) necessary for mediating the endoplasmic reticulum rearrangement. Readthrough (RT) of ORF1 allows expression of an 82 kDa protein (RT-ORF1). Both 23kDa and 82 kDa proteins are virus replication associated components. Three overlapping small ORFs (p7a, p7b and p5a) in the central region of BBSV genome are necessary for cell-to-cell movement. A 24.5 kDa protein (p24) corresponding to coat protein (CP) is encoded by ORF6. The N-terminal basic amino acids and phosphorylation of the CP are critical for virion assembly and virus systemic movement [10].

Research on the genetic diversity of viruses provided critical information for understanding virus evolution, geographical origin, virulence variations, and the occurrence of emerging new epidemics. Genomic variation caused by mutation in plant RNA viruses is further enhanced by recombination, reassortment, and acquisition of extra genomic components. RNA genome generally has a higher mutation ratio therefore each RNA virus isolate is a cloud of related mutants (quasispecies) and the frequency of each of the mutants in the cloud changes in response to selection pressure [11].

In this study, complete genome sequences of four BBSV isolates originally collected from North-East part of Iran was sequenced and analyzed together with those for BBSV isolates previously reported to achieve information on the biogeography, evolution, and spread of the virus. Recombination is a main source of diversification in many plant RNA and DNA viruses by generating genetic variation, reducing mutational load and producing new viruses [12, 13]. Recombination analysis indicates that a genomic exchange is responsible for the emergence of new BBSV strains, and provides new information for better understanding of the diversity and evolution of betanecroviruses. Analysis of selective pressure on the proteins encoded by the BBSV genome indicates that amino acid substitutions are also involved in BBSV evolution. This analysis could also increase our understanding of protein structure and function along with virus epidemiology. The high fixation index (*F*_*ST*_) values indicate large genetic differentiation among the BBSV populations, suggesting more than one origin (unlike BNYVV). Furthermore, our results showed no indication of frequent gene flow between populations in Mid-Eurasia, East-Asia and European countries.

## Materials and methods

### Virus isolates

**Fig 1:**
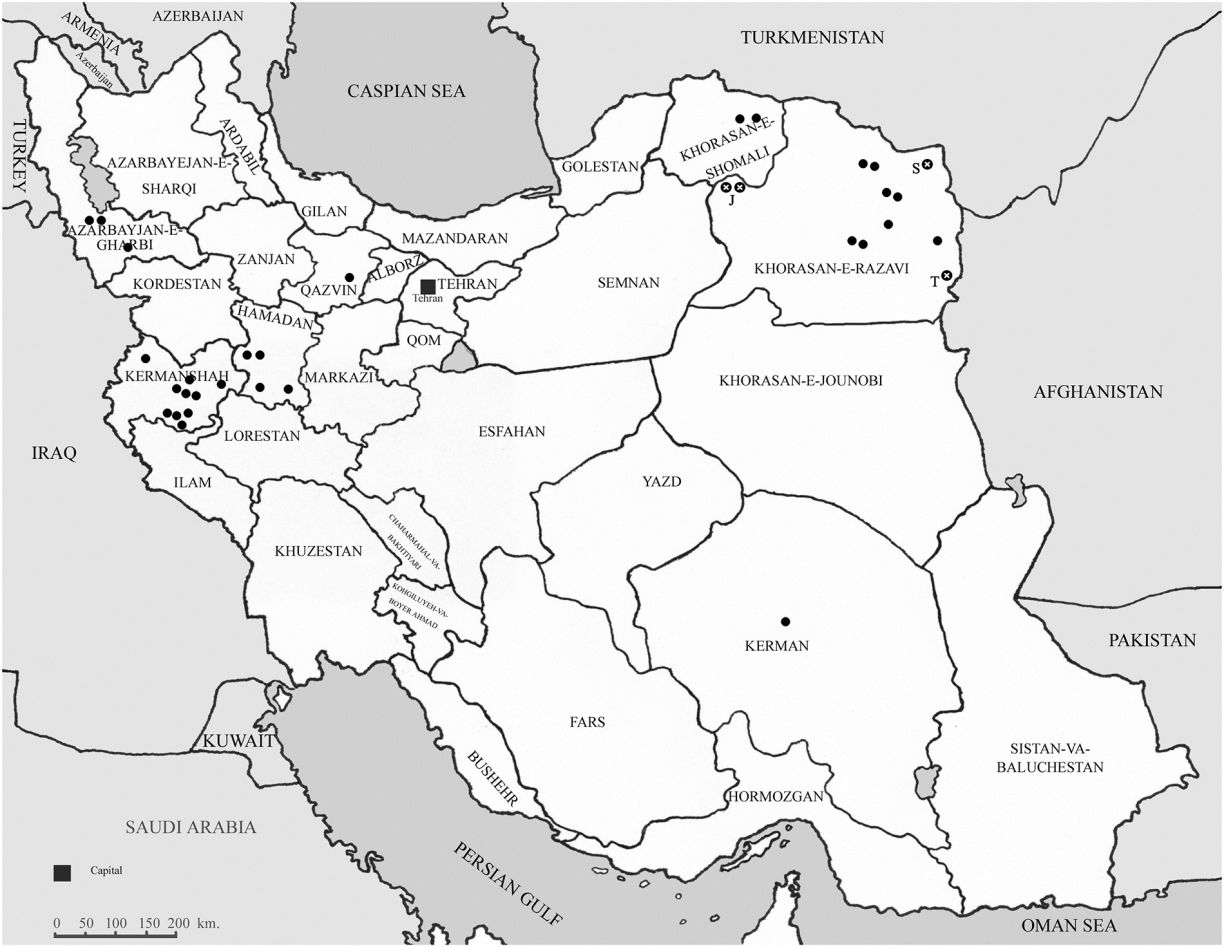
Map of Iran showing the origin of all Iranian *Beet black scorch virus* isolates (marked by black circle). Location of the three counties surveyed, Jovein, Sarakhs, and Taibad are shown by “J”, “S” and “T”, respectively. Locations of four isolates sequenced in this study are marked by black circle with cross mark.

Soil samples were collected from 10 sugar beet fields in three counties (Jovein, Sarakhs, and Taibad) of Khorasan Razavi province in Iran (Table A in S1 File) and were assayed for BBSV in the greenhouse. An autoclaved potting soil was used as control. Soil samples were mixed with equal parts of autoclaved sand to facilitate roots removal from susceptible *B*. *vulgaris* cv. Jolge plants at harvest. Soil samples were placed in new 280-ml cups (used instead of pots) with holes in the bottom for drainage. The cups were placed in sterilized plastic saucers spaced on greenhouse benches to avoid cross contamination due to water splash. The plants were harvested 8–10 weeks after planting and used for nucleic acid extraction and sequencing. Each sequence obtained represents a single virus isolate. The isolates used in this study were selected from soil samples collected from ten various sugar beet fields (Fig 1).

### Enzyme-linked immunosorbent assay (ELISA)

Samples were prepared following washing the roots of seedlings from each pot to remove the soil. Root tissue samples (0.2 g from each root mass) was placed in extraction bags containing 2 ml of extraction buffer (0.05 M phosphate-buffered saline, pH 7.2, 0.5% Tween 20, 0.4% dry milk powder) and homogenized with a handheld roller press. Extracted sap was added to duplicate wells of a microtiter plate (100 μl per well). Each plate also contained controls including sap from BBSV-infected and healthy beet roots. Double antibody sandwich ELISA reagents were purchased from LOEWE (Sauerlach, Germany) and were used to assay for BBSV. Purified IgG prepared against BBSV (1 mg/ml) was used to coat microtiter plates at a dilution ratio of 1:200 according to the manufacturer’s instructions. Alkaline phosphatase-conjugated anti-BBSV IgG was added to wells (dilution 1:200). Alkaline phosphatase substrate (Sigma Chemical Co., St. Louis, MO) was used at a ratio of 5 mg in 8.3 ml of substrate buffer. Absorbance readings (*A*405nm) were recorded 30 min after the addition of substrate using a Bio-Tek ELx800 microplate reader (Winooski, VT). Absorbance values greater than the mean plus three times the standard deviation of the OD test values of the negative controls at 405 nm were considered positive.

### Immuno-capture reverse transcription-polymerase change reaction (IC-RT-PCR)

Immuno-capture of BBSV virions was performed in 0.2 mL PCR tubes pre-coated with 50 μL of virus-specific IgG (2 μg/mL, in carbonate buffer pH 9.6), incubated for 3 hr at 37°C and subsequently washed three times with PBS-Tween. Then 50 μL of sap extract (0.1 g of root extract in 1 mL of PBS-Tween) was added to the tube and incubated at 4°C overnight, followed by two washes with PBS-Tween and one wash with DEPC-treated water. Complementary DNA (cDNA) was prepared in a final volume of 20 μL. For complementary DNA (cDNA) synthesis, 1 μL of reverse primer (2587 5′-CTCCAATAGTTATGTATTGCGTCTTC-3′ 2561) (20 pmol/ μL), 2 μL of dNTPmix (10 mM) and 3 μL of DEPC-treated water were added to the tube, heated at 65°C for 5 min and immediately chilled on ice. A mixture of 2 μL of 10x first-strand buffer [250 mM Tris-HCl (pH 8.3), 375 mM KCl, 15 mM MgCl2], 2 μL of DTT (0.1 M), 1 μL of RNase inhibitor (40 U) and 200 U of M-MuLV were added to the tubes. The tubes were heated at 25°C for 10 min and then at 42°C for 50 min. For the PCR, 5 μL of the cDNA was placed in a new tube and added 5 μL of 10x PCR Buffer [200 mM Tris-HCl (pH 8.4), 500 mM KCl], 3 μL of MgCl2 (50 mM), 2 μL of dNTP mix (10 mM), 2 μL of reverse and forward primers (2063 5′-ACAATCCCGCTACTCATTTTGGCGTG-3′ 2088) (10 μM), 1 μL of *Taq* DNA polymerase (5 U) and 30 μL of DEPC-treated water. The cycling profile consisted of an initial denaturing step at 94°C for 3 min followed by 35 cycles of 94°C for 3 min, 51.5°C for 90s and 72°C for 60s; and a final extension step at 72°C for 10 min. The amplification products were cloned into pGEM-T Easy vectors (Promega, Madison, WA, USA) and used to transform DH5α *Escherichia coli* competent cells according to the manufacturer’s instructions. Recombinant plasmids were extracted using QIApreb Spin Miniprep Kit (QIAGEN, Valencia, CA, USA) and purified for nucleotide sequencing. The nucleotide sequence numbering refers to the Iranian isolate Iran-Ksh1 (accession no. FN543421).

### PCR, cloning, and sequencing

The full-length genome sequences of BBSV isolates were amplified by RT-PCR from the total RNA extracted from infected sugar beet roots using Tri-reagent (Sigma, USA) and first-strand cDNA was synthesized using M-MuLV reverse transcriptase (Fermentas, Lithuania), according to the manufacturer’s instructions. The full-length genome of BBSV isolates was amplified using three pairs of primers (BBF1 5′-AAGAAACCTAACCAGTTTCTCGTTGA-3′ and BBR3 5′-TTGCATCTCCATGCCAGCCTGATC-3′); (BBF3 5′-TGCTGAGGAACATCTGTTCGA-3′ and BBR5 5′-CATTTCAGAAGTGGAAATGTTGTGT-3′) and (BBF5 5′-AAGAARGAYATGGGTCCATCGG-3′ and BBR7 5′-GGGCACCTGGAAYACCAGGTAT-3′) with at least 50–100 nt overlap. Purified RT-PCR generated DNA fragments using the QIAquick Gel Extraction Kit (QIAGEN, Valencia, CA, USA) were used as templates for direct sequencing or cloned into *Eco*RV site of plasmid pZErO-2 (Invitrogen, Carlsbad, CA, USA), transferred into *E*. *coli* strain DH5α and plated on media containing 25 μg ml^-1^ kanamycin. Plasmids were extracted using QIApreb Spin Miniprep Kit (QIAGEN, Valencia, CA, USA). PCR products or cloned fragments were sequenced by a primer walking approach in both directions using the BigDye Terminator version 3.1 Cycle Sequencing Ready Reaction Kit (Applied Biosystems) and an Applied Biosystems Genetic Analyser DNA Model 310. For sequencing of each PCR fragment different forward and reverse primers with 50 to 100 nucleotides apart were designed. Overlapping sequences sharing 99–100% nucleotide identity were assembled to ensure that they came from the same genome and not from different components of a genome mixture. Nucleotide sequences of the cloned fragments for each isolate were determined using three to five cDNA clones and when any differences was detected, then its RT-PCR products were directly sequenced to determine which was the most common and majoritarian one. Sequence data were assembled using BIOEDIT version 5.0.9 [14]. The sequences were compared with other sequences in the GenBank by the BLAST program of the National Center for Biotechnology Information (NCBI).

### Phylogenetic relationship and estimation of genetic distances

The full-length genome sequences of four BBSV isolates obtained in this study and all available BBSV sequences in the GenBank were used for phylogenetic analyses (Table B in S1 File). Non-overlapping regions of the overlapping ORFs were used for genetic and population analysis. Phylogenetic tree for each ORF and 3′UTR were estimated using the Maximum-Likelihood (ML) method in MEGA6 [15]. We estimated the model of nucleotide substitution that best fitted the data using the application BestModelTest implemented in the MEGA6. For the ML analysis, we used the Kimura’s two-parameter (K2) model of nucleotide substitution with rate variation among sites modeled using a gamma distribution and a proportion of invariable sites (K2+I+G). The signals for virus replication are located in the promoters at the 3′UTR of the plus and minus strands therefore phylogenetic tree using 3′UTR is shown. Branch support was evaluated by bootstrap analysis based on 1000 pseudoreplicates. The ML trees were compared using PATRISTIC [16]. Nucleotide distances and nucleotide diversity (mean nucleotide distance between two randomly selected sequence variants) were estimated by the maximum-composite-likelihood method with MEGA6 [15]. Pairwise synonymous substitutions per synonymous site (*dS*) and nonsynonymous substitutions per nonsynonymous site (*dNS*) were also calculated according to the Pamilo-Bianchi-Li (PBL) method based on Kimura’s two-parameter model [17]. Standard deviations were calculated by the bootstrap method with 1000 repeats. Furthermore, pairwise genetic distances were analyzed by the Kimura’s two-parameter method implemented in Phylip 3.67 software [18] for each gene and 3′UTR. DNASP version 4.10 [19] was used to estimate haplotype diversity. Haplotype diversity was calculated based on the frequency and number of haplotype in the population.

### Detection of recombination

Relationship between aligned genes, 3′UTR, and full-length genome sequences (Table B in S1 File) were calculated separately using Maximum Likelihood (ML) method implemented in MEGA6 [15]. Recombination events, major and minor parental isolates of recombinants, and recombination break points were analyzed using several methods implemented in the RDP4 version 4.70 [20] with default configuration and Bonferroni corrected *P*-value cut-off of 0.05 and 0.01. Putative recombinants found by the RDP4 were confirmed using SISCAN version 2.0 [21].

### Selection analyses

A ML method has been developed for detecting amino acids under positive selection [22]. This method originally employed 14 models that use statistical distributions to account for variable *ω* (*dNS*/*d*S) ratios among codon sites. But models M0, M1, M2, M3, M7, and M8 are sufficient for accurate selection analysis [23]. Models M0, M1 and M7 do not allow for the existence of positively selected sites. M0 calculates a single *ω* ratio (between 0 and 1) averaged over all sites, M1a (nearly neutral) account for neutral evolution by estimating the proportion of conserved (*ω* = 0) and neutral (*ω* = 1) sites, whereas M7 uses a discrete β distribution (between the same bounds) to model different *ω* ratios among sites. The shape of the beta distribution is governed by the parameters *p* and *q*, alternatively models M2, M3 and M8 account for positive selection using parameters that estimate *ω* > 1. Models M2 and M8 extend M1 and M7, respectively, through the addition of two parameters (*p*_2_ and *ω*_2_ for M2 and *p*_1_ and *ω*_1_ for M8) that have the potential to estimate *ω* > 1 for an extra class of sites. M3 provides the most sensitive test for positive selection by estimating a *ω* ratio for a predetermined number of classes. Three classes were used in this analysis (*p*_0_, *p*_1_, and *p*_2_) such that three corresponding *ω* ratios (*ω*_0_, *ω*_1_ and *ω*_2_) were estimated. The first step in the identification of amino acid sites under positive selection is to test whether sites exist with *ω* > 1 by comparing nested models using likelihood ratio tests (LRTs). M0 and M1 are both special cases of M2 and M3, while M7 is a special case of M8, and such nested models can be compared with LRTs. Three LRTs (M3 vs M0, M2a vs M1a and M8 vs M7) were used to assess the models’ fit to the data, as described by Wong et al. [24]. Once positively selected sites have been shown to exist, the second step is to use Bayesian methods to locate their position. Sites having high posterior probabilities (> 90%) of belonging to a site class with *ω* > 1 are good candidates for positively selected sites. Posterior probabilities are conditional on the observed data such that they refer to the probability that a site, given the data at that site, is from a particular site class. The methods and models described here were implemented using the CODEML program of the PAML package, version 3.0c [25].

## Results

### Sequence analysis and phylogenetic relationships

**Fig 2:**
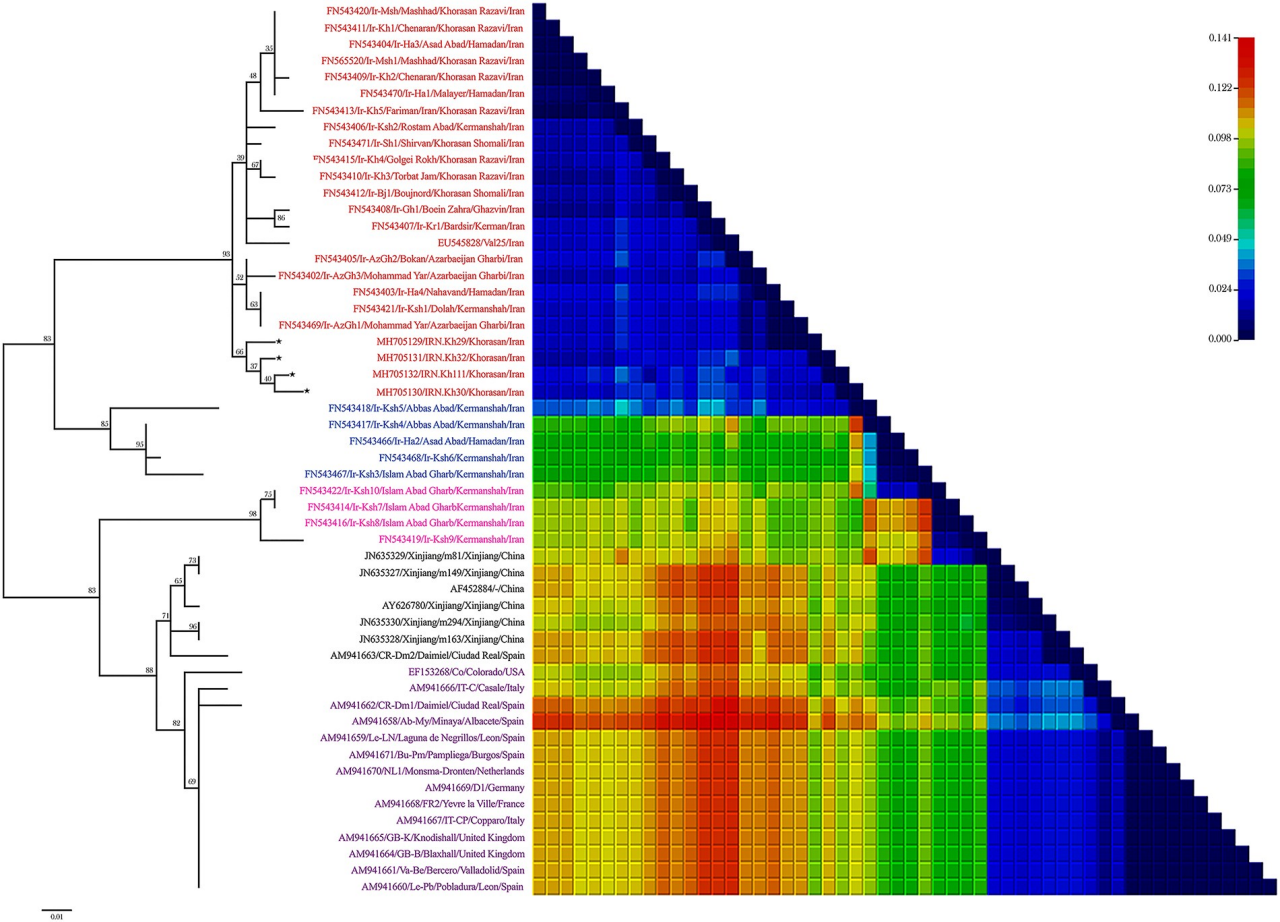
Maximum Likelihood (ML) tree and two dimensional nucleotide diversity plot showing the relationship among *Beet black scorch virus* isolates. The tree was constructed using fifty four 3′UTR nucleotide sequences of BBSV isolates. Numbers at each node indicates the percentage of supporting puzzling steps (or bootstrap samples) in ML method. The name of each isolate and the country of its origin are listed in the accession number in the International Gene Sequence Database (GenBank). The isolates sequenced in this study are marked by star mark. Subgroups were highlighted by red, blue, pink, black and purple for I-IranA, I-IranB, II-IranC, II-Chinese, and II-Europe, respectively.

A total of 172 beet root samples were tested by DAS-ELISA of which thirty-three samples (19.2%) were tested positive for BBSV. Per cent of virus infected plants varied among collection regions. The highest percentage of BBSV-infected plants was found in Jovein county (17 infected/65 plants tested, 26.15%), followed by Taibad (10/53: 18.86%), and Sarakhs (6/54: 11.11%) (Table A in S1 File). The nucleotide sequence of four BBSV isolates (GenBank accessions no: MH705129 to MH705132) corresponding to full-length genome were determined. All genomes were 3644 nucleotides in length had the typical BBSV genome organization with six ORFs. For ORF1, RT-ORF1, ORF3, ORF4, ORF5, and ORF6, the length of the nucleotide sequences were 36–647 (RNA polymerase), 36–2210 (produced by translational readthrough of ORF1), 2228–2419 (protein A, p7a), 2421–2618 (protein B, p7b), 2434–2577 (protein A, p5a), and 2647–3345 (CP, p24), respectively. The gene sequence of the IRN-Kh29 isolate was used to search the GenBank database using the BLAST program. The search showed the nucleotide sequence identities for ORF1, RT-ORF1, ORF3, ORF4, and ORF6 genes were 87–98%, 89–99%, 92–99%, 91–99%, and 85–99%, respectively. Pairwise comparisons using CLUSTALX2 were performed for RT-ORF1 ORF3, ORF4, ORF6 and 3′UTR regions. The phylogenetic trees based on all ORFs (Figures A, B and C in S2 File) indicted that BBSV isolates fell into two main groups. In addition, using 3′UTR sequences, BBSV isolates clearly divided into groups GI and GII which further subdivided into five subgroups (Fig 2). Most of the Iranian isolates clustered in GI with two subpopulations IranA (I-IranA) and IranB (I-IranB). Furthermore, four Iranian BBSV isolates from North-West (Ir-Ksh7, Ir-Ksh8, Ir-Ksh9, and Ir-Ksh10) were grouped in the distinct subgroup IranC in GII (II-IranC). All Chinese isolates and one isolate from Spain (CR-Dm2) fell into subgroup Chinese from GII (II-Chinese), whereas the European isolates together with the USA isolate clustered in subgroup Europe (II-Europe) (Fig 2). Two-dimensional pairwise nucleotide distances plot analysis also showed two main phylogenetic Groups. BBSV isolates in GI are closely related to each other which confirmed by low nucleotide diversity (0.000 to 0.073; high similarities). However, high pairwise nucleotide distances 0.073 to 0.141 were indicated for GII (Fig 2). The pairwise nucleotide distances for each subgroup were 0.000 to 0.049 (I-IranA); 0.049 to 0.073 (I-IranB); 0.073 to 0.098 (II-IranC); 0.098 to 0.122 (II-Chinese); and 0.122 to 0.141 (II-Europe) (Fig 2).

### Patristic distance plots

**Fig 3:**
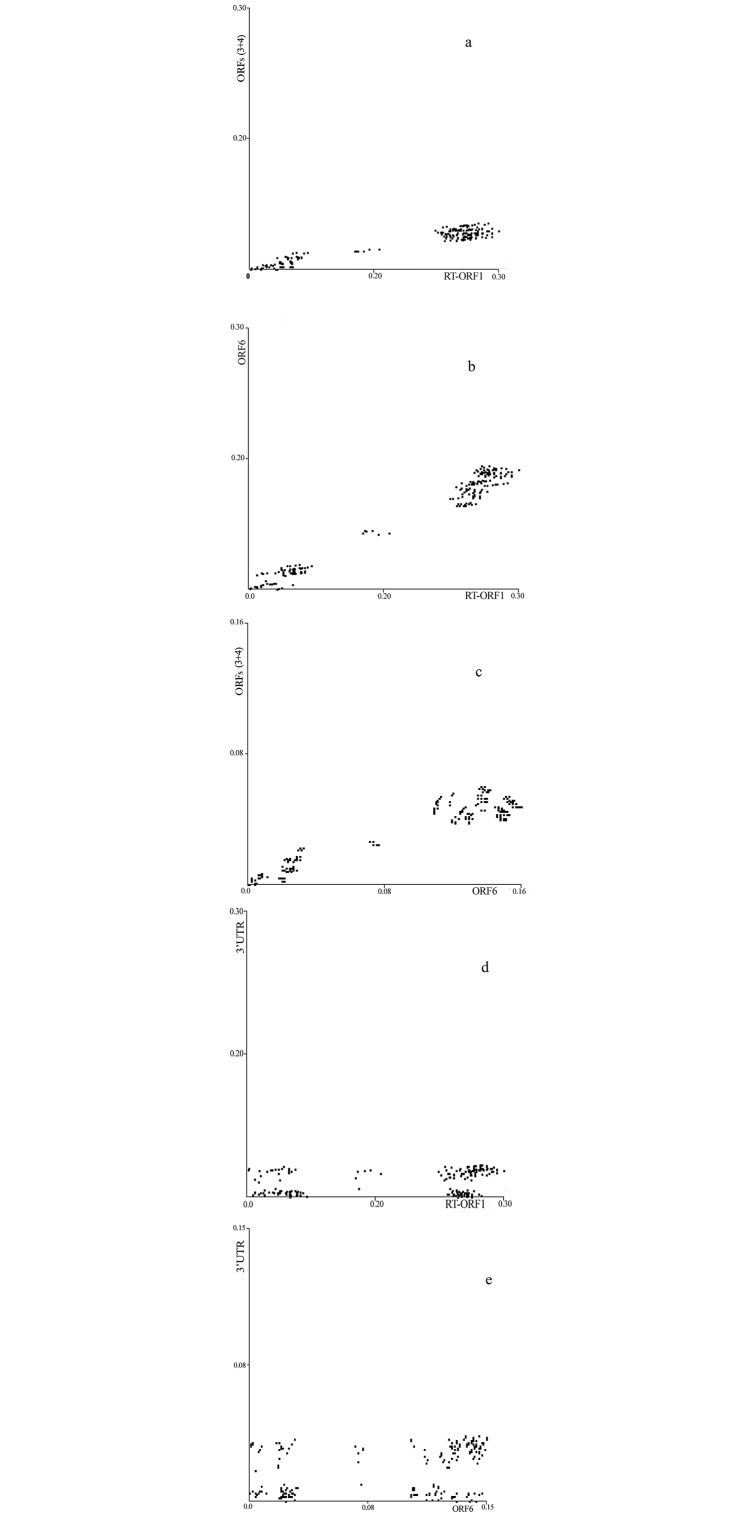
Multidimensional scaling of tree-to-tree patristic distances (a) ORFs (3+4) vs RT-ORF1 isolates; (b) ORF 6 vs RT-ORF1 isolates; (c) ORFs (3+4) vs ORF6 isolates; (d) 3′UTR vs RT-ORF1 isolates; and (e) 3′UTR vs ORF6 isolates.

We constructed pairwise comparison of the maximum-likelihood trees of the distinct ORFs by PATRISTIC approach. All pairwise plots of the distances in the trees deduced from the RT-ORF1 versus each ORF3, ORF4, and concatenate ORFs (3+4) showed similar templates. This is demonstrated by the plot of ORFs (3+4) against RT-ORF1 distances (Fig 3a), in which the three sets of distances show a linear correlation coefficient of 0.959 (p<0.001). The plot of the RT-ORF1 distances against ORF6 (CP) with a linear correlation coefficient of 0.986 (Fig 3b) and plot of the ORFs (3+4) distances against ORF6 with a linear correlation coefficient of 0.944 (Fig 3c) have a similar pattern with three distinct subpopulations. The pairwise plots of the 3′UTR vs ORFs (RT-ORF1, Fig 3d and ORF6, Fig 3e) were constructed and showed three subpopulations but the linear correlation coefficients were less than those of ORFs vs ORFs. No correlation was detected between 3′UTR and concatenated fragments of ORFs (3+4).

### Recombination analysis

**Fig 4:**
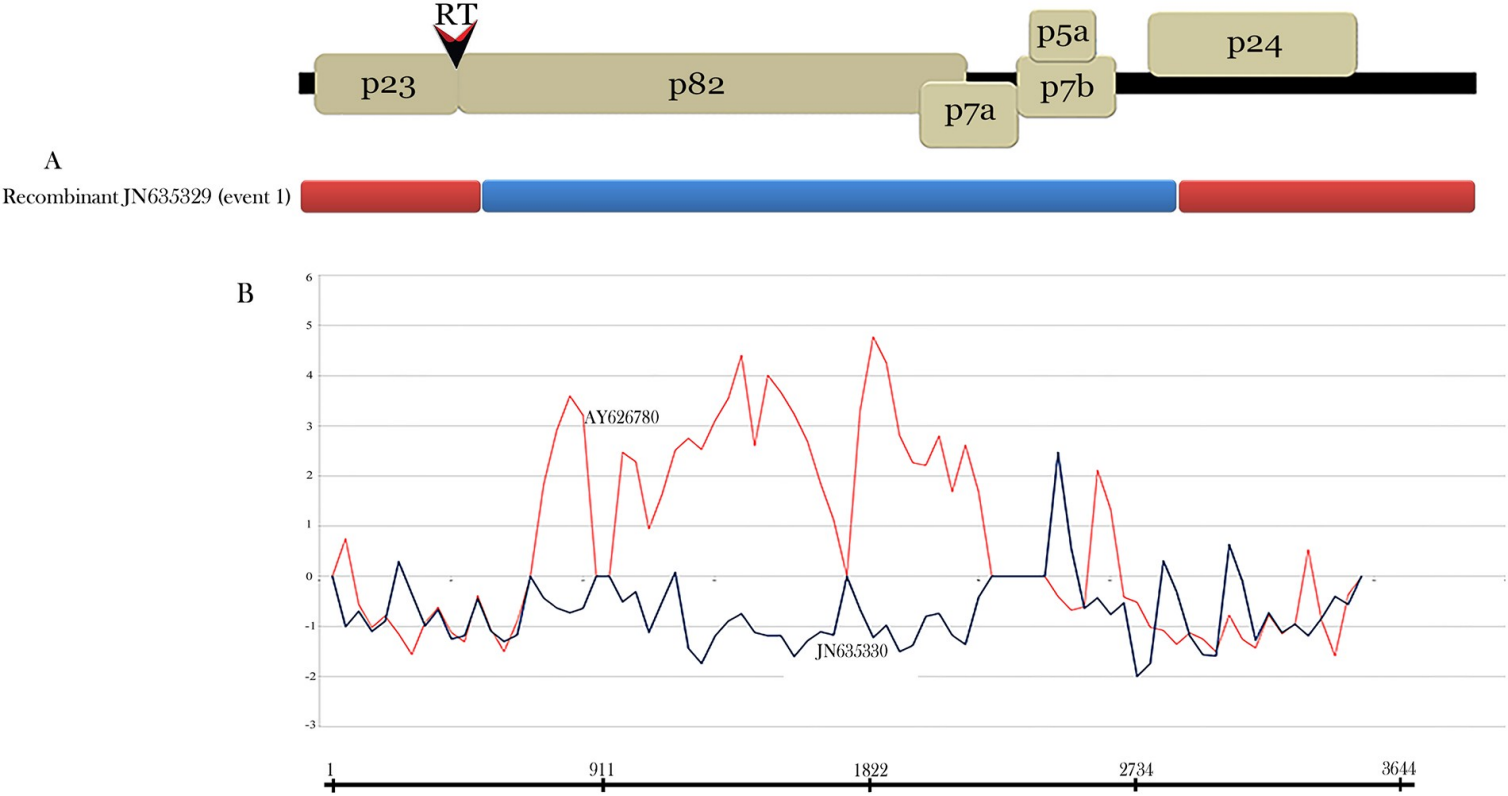
The genome organization of *Beet black scorch virus* is shown at the top of the figure. (A) The schematic recombination event 1 is shown for isolate Xinjiang/m81 (JN635329). Major and minor parents are indicated by Red and Blue boxes. (B) Graph showing SISCAN v.2 analysis of the recombinant Xinjiang/m81 isolate (JN635329) with the Xinjiang (AY626780) (Red color) and Xinjiang/m294 (JN635330) (Blue color) as the putative major and minor parents, respectively.

Different methods were used for recombination breakpoint prediction that provided evidence for intra and inter recombination events across the BBSV genomes analyzed. Recombination analysis found that the Xinjiang/m81 sequence had two recombination sites around nucleotide 668 in the RT-ORF1 and nucleotide 2714 in the ORF6 genes (event 1). This is a ‘clear’ intra-recombinant of Xinjiang and Xinjiang/m294 isolates in subgroup II-Chinese as putative parents. Putative parental isolates are referred to nonrecombinant sequences. These include regions that are most closely related to those of the recombinant sequences, showing the lineages that most probably provide those regions of the recombinant genomes. Recombination sites were detected by *P*-values using the RDP (2.738×10^−6^), GENECONV (4.413×10^−6^), BootScan (1.424×10^−3^), Maxchi (4.585×10^−10^), Chimaera (1.465×10^−5^) and Siscan (4.348×10^−16^) programs of the RDP4 software, and SISCAN v. 2 program in the original software (Fig 4A and 4B, Table 1). In addition, a putative inter-recombination breakpoint (event 2) was detected using RDP4 in 3′UTR region of Iranian isolates in subgroup I-IranA (Figure D in S2 File, Table C in S1 File) with the likely parental isolates Ir-Ksh9 (FN543419) belonging to subgroup II-IranC and Ir-Ksh5 (FN543418) from subgroup I-IranB, as major and minor parents, respectively. However, the recombination event 2 was not supported with a high degree of confidence (with multiple different methods and with a low associated *P*-value for each of the methods). The RDP4 results are shown for two putative recombinant isolates Ir-Kr1 and Ir-Gh1 in Table 1 which detectable by Siscan program. No recombination site was found in the ORF3 and ORF4, which are involved in cell to cell movement of the virus.

**Table 1:** Crossover sites in *Beet black scorch virus* isolates detected using recombination detecting programs.

Event	Recombinant (Ac. No)	Parental isolates (Ac. No.) ^a^		Breakpoints ^b^ /ORF		Methods ^c^						SISCAN (*Z*-value) ^d^
		Major parent	Minor parent	Begin	End	RDP	GENECONV	BootScan	Maxchi	Chimaera	Siscan	
1	Xinjiang/m81 (JN635329)	Xinjiang (AY626780)	Xinjiang/m294 (JN635330)	668/RT-ORF1	2714/ORF6	2.738×10^−6^	4.413×10^−6^	1.424×10^−3^	4.585×10^−10^	1.465×10^−5^	**4.348×10**^**−16**^	3.6
2	Ir-Kr1 (FN543407)	Ir-Ksh9 (FN543419)	Ir-Ksh5 (FN543418)	ND ^e^	3539/3′UTR	ND	1.820×10^−1^	7.896×10^−2^	2.974×10^−2^	ND	**9.313×10**^**−7**^	ND
	Ir-Gh1 (FN543408)	Ir-Ksh9 (FN543419)	Ir-Ksh5 (FN543418)	ND	3539/3′UTR	0.104	1.540×10^−1^	6.323×10^−2^	5.734×10^−2^	1.031×10^−2^	**3.535×10**^**−6**^	ND

^a^ Parental isolate means the most likely isolate among analyzed isolates.

^b^ Numbers indicate recombination sites.

^c^ Recombination break points were analyzed using RDP, GENCONV, BOOTSCAN, MAXCHI, CHIMAERA and SISCAN methods, in the RDP4 v.4.70 [20]. The highest *P*-value presented was determined by the method indicated in bold type.

^d^ The *Z*-value was calculated by original SISCAN v.2 program. The location of recombination sites were determined based on the sequence of the Xinjiang isolate (AY626780).

^e^ Not determined.

### Mean nucleotide diversity and selection analysis

The mean nucleotide diversities for the RT-ORF1, ORF3, ORF4 and ORF6 were 0.089, 0.056, 0.071 and 0.141, respectively (Table 2). In addition, the within-group diversity of BBSV genes was less, from 0.008 to 0.042 (Table 2). We also estimated pairwise *dNS*/*dS* ratios using the PBL method [16]. When all isolates were included, the highest and lowest *dNS*/*dS* ratios were 0.147 for ORF4 and 0.020 for ORF3 (Table 2).

**Table 2:** Nucleotide and haplotype diversity of four ORFs of *Beet black scorch virus* isolates ^a^.

ORFs	Phylogenetic groups	*D*^b^	Haplotype diversity	*d*_*S*_	*d*_*NS*_	*d*_*NS*_/*d*_*S*_
RT-ORF1	I-IranA	0.018 (0.002)	1.000	0.052 (0.006)	0.003 (0.001)	0.057
	I-Iran-B	0.006 (0.001)	1.000	0.008 (0.002)	0.004 (0.001)	0.500
	II-Chinese	0.031 (0.002)	1.000	0.090 (0.008)	0.006 (0.001)	0.067
	All isolates	0.089 (0.004)		0.298 (0.017)	0.015 (0.002)	0.050
ORF3	I-IranA	0.016 (0.006)	0.955	0.050 (0.030)	0.005 (0.003)	0.100
	I-Iran-B	0.007 (0.005)	0.666	0.022 (0.016)	ND^c^	ND
	II-Chinese	0.015 (0.005)	0.905	0.043 (0.018)	0.005 (0.003)	0.116
	All isolates	0.056 (0.013)		0.197 (0.050)	0.004 (0.001)	0.020
ORF4	I-IranA	0.015 (0.006)	0.977	0.034 (0.018)	0.006 (0.005)	0.176
	I-Iran-B	0.010 (0.006)	0.666	ND	0.013 (0.008)	ND
	II-Chinese	0.019 (0.006)	0.809	0.029 (0.015)	0.014 (0.007)	0.482
	All isolates	0.071 (0.015)		0.190 (0.059)	0.028 (0.010)	0.147
ORF6	I-IranA	0.028 (0.004)	1.000	0.082 (0.013)	0.004 (0.002)	0.048
	I-Iran-B	0.006 (0.002)	1.000	0.009 (0.006)	0.005 (0.003)	0.555
	II-Chinese	0.045 (0.006)	0.952	0.145 (0.019)	0.005 (0.002)	0.034
	All isolates	0.141 (0.012)		0.556 (0.055)	0.018 (0.004)	0.032

^a^ Substitutions: *dNS* = average number of nonsynonymous substitutions per nonsynonymous site, *dS* = average number of synonymous substitutions per synonymous site, and *dNS/dS* = average of the ratio between nonsynonymous and synonymous substitutions.

^b^ *D* = nucleotide diversity: average number of nucleotide substitutions per site between all pairs of sequences in the group. Standard errors are indicated in parentheses.

^c^ ND = Not determined.

ML method implemented in PAML [22] was used to find variations in the *ω* ratio between sites. This method enables detection of distinct codon sites under positive selection and eliminates the other hypothesis about population demography correlated with other statistical tests of selection. Generally, the evolutionary constraint applied on ORF3 is larger than the one exerted on other ORFs and no site was detected under positive selection (Table 3).

**Table 3:** The *dNS/dS* (*ω*) values, log-likelihood (ln*L*) values, likelihood ratio test (*χ*^*2*^) statistics and positively selected amino acid sites undergoing different models of codon substitution used to investigate selection pressures on three proteins encoded by the *Beet black scorch virus* genome analyzed in this study.

Protein	Models^1^	Parameter estimates	*ω* ratio	ln*L*	*χ*^*2*^ ^2^	Positively selected (amino acids) sites ^3^	
						NEB	BEB
RT-ORF1	M0	*ω* = 0.056	0.056	-6032.784		None	
	M3	*p0* = 0.114, *p1* = 0.870, *p2* = 0.014, *ω0* = 0.000, *ω1* = 0.048, *ω2* = 1.223	0.060	-6016.595	*P* < 10^−6^	15V [0.942*], 39L [0.829], 188Q [1.000**], 461T [0.980*], 641S [0.559], 672A [0.633], 711I [0.973*]	
	M1a	*p0* = 0.981, *p1* = 0.018, *ω0* = 0.041, *ω1* = 1.000	0.059	-6016.679		Not allowed	
	M2a	*p0* = 0.984, *p1* = 0.000, *p2* = 0.015, *ω0* = 0.042, *ω1* = 1.000, *ω2* = 1.179	0.060	-6016.603	*P* = 0.927	15V [0.958*], 17H [0.551], 39L [0.853], 152T [0.505], 188Q [1.000**], 461T [0.985*], 641S [0.612], 672A [0.680], 711I* [0.980]	15V [0.511], 39L [0.583], 188Q [0.726], 461T [0.619], 711I [0.588]
	M7	*p* = 0.230, *q* = 3.443	0.058	-6020.971		Not allowed	
	M8	*p0* = 0.985, *p* = 4.525, *q* = 99.0, *p1* = 0.014, *ω* = 1.234	0.060	-6016.598	*P* = 0.012	15V [0.929], 39L [0.821], 188Q [1.000**], 461T [0.975*], 641S [0.545], 672A [0.620], 711I* [0.967]	15V [0.672], 39L [0.738], 188Q [0.944], 461T [0.826], 672A [0.530], 711I [0.789]
ORF3	M0	*ω* = 0.040	0.040	-422.611		None	
	M3	*p0* = 0.179, *p1* = 0.397, *p2* = 0.423 *ω0* = 0.048, *ω1* = 0.048, *ω2* = 0.041	0.040	-422.719			
	M1a	*p0* = 1.000, *p1* = 0.000, *ω0* = 0.048, *ω1* = 1.000	0.040	-422.611		Not allowed	
	M2a	*p0* = 1.000, *p1* = 0.000, *p2* = 0.000, *ω0* = 0.048, *ω1* = 1.000, *ω2* = 1.000	0.040	-422.611			
	M7	*p* = 5.137, *q* = 99.00	0.041	-422.617			
	M8	*p0* = 1.000, *p* = 5.136, *q* = 99.00, *p1* = 0.000, *ω* = 3.010	0.041	-422.724			
ORF4	M0	*ω* = 0.229	0.229	-479.925		None	
	M3	*p0* = 0.584, *p1* = 0.387, *p2* = 0.028 *ω0* = 0.171, *ω1* = 0.171, *ω2* = 3.622	0.268	-477.700	*P* = 0.348	8Q [0.976*]	
	M1a	*p0* = 0.868, *p1* = 0.131, *ω0* = 0.115, *ω1* = 1.000	0.231	-478.055		Not allowed	
	M2a	*p0* = 0.971, *p1* = 0.000, *p2* = 0.028, *ω0* = 0.171, *ω1* = 1.000, *ω2* = 3.625	0.268	-477.543	*P* = 0.599	8Q [0.976*]	8Q [0.768]
	M7	*p* = 0.246, *q* = 0.771	0.241	-478.469			
	M8	*p0* = 0.972, *p* = 20.68, *q* = 99.00, *p1* = 0.027, *ω* = 3.665	0.269	-477.703	*P* = 0.128	8Q [0.973*]	8Q [0.851]
ORF6	M0	*ω* = 0.041	0.041	-2236.821		None	
	M3	*p0* = 0.709, *p1* = 0.281, *p2* = 0.008, *ω0* = 0.000, *ω1* = 0.125, *ω2* = 3.838	0.066	-2222.959	*P* < 10−^6^	12S [0.889], 155R [0.981*]	
	M1a	*p0* = 0.971, *p1* = 0.028, *ω0* = 0.026, *ω1* = 1.000	0.053	-2226.188		Not allowed	
	M2a	*p0* = 0.973, *p1* = 0.022, *p2* = 0.003 *ω0* = 0.027, *ω1* = 1.000, *ω2* = 8.884	0.078	-2225.783	*P* = 0.667	155R [0.761]	155R [0.899]
	M7	*p* = 0.135, *q* = 2.549	0.045	-2225.159		Not allowed	
	M8	*p0* = 0.992, *p* = 0.208, *q* = 5.011, *p1* = 0.007, *ω* = 4.081	0.067	-2222.998	*P* = 0.115	12S [0.775], 155R [0.972*]	12S [0.576], 155R [0.961*]

^1^ Model descriptions is according to [22] M0 (one ratio); M3 (discrete); M7 (β); M8 (β plus *ω*), [24], M1a (nearly neutral); M2a (positive selection).

^2^ Likelihood ratio tests (LRTs) are performed by taking twice the difference in log likelihood between two models and comparing the value obtained with a *χ*^*2*^ distribution (degrees of freedom equal to the difference in the number of parameters between the models). Degrees of freedom are 2 for the M2a vs M1 and M7 vs M8 comparisons, and 4 for the M0 vs M3 comparisons. *p*-values indicate comparisons where the null hypothesis can be rejected in regards to the alternative hypothesis.

^3^ Amino acid (codon) sites undergoing positive selection are shown. Identification of positively selected amino acids is based on either the Naive Empirical Bayes (NEB) approach (under M3) or the Bayes Empirical Bayes (BEB) approach (with the M2a, M8, and branch-site model A). The posterior probabilities for each amino acid under positive selection is shown in the bracket and those with higher posterior probabilities of P>95.0 and P>99.0 are indicated by [*] and [**], respectively.

The model M0 was used to evaluate selection pressures [maximum likelihood (ML) framework of codon substitution]. The selection pressure values obtained were 0.056, 0.040, 0.229 and 0.041 for RT-ORF1, ORF3, ORF4 and ORF6, respectively (Table 3). Three models (M2a, M3, and M8) predicted a positively selected group of sites in the polymerase gene (RT-ORF1). M3 was not restricted in this way and estimated that 1.4% of sites are under weak positive selection (*ω*_2_ = 1.223). In addition, M8, which also estimated a small group of sites with a similar positive selection pressure (*ω*_1_ = 1.896) was able to reject M7 and approved the significance of positive selection in RT-ORF1. Using Bayesian methods, M2, M3, and M8 showed that sites 15V, 188D, 461T, and 711T belong to the positively selected class with posterior probabilities > 90% (Table 3). Although M3 could not reject M2 in a likelihood ratio test (LRT), sites 39L, 641S, and 672A should still be under possible positive selection because they were also detected by M8 (Table 3). A positive selection site was found in the ORF4 data set using M2, M3, and M8, which rejected models M0, M1 and M7 in LRTs (Table 3). M2 and M3 showed similar results and predicted a large set of conserved sites and a small set of positively selected sites (*ω* > 2). By Bayesian methods, three models indicated that the amino acid 8Q was under positive selection with posterior probability of > 95. No site was detected under positive selection for ORF3 (p7a). The model M0 predicted a similar likelihood in comparison to other models (as described above) which were to detect positive selection and proposed that the sites on ORF6 (CP) are strongly conserved (*ω* = 0.041). However, values indicating positive selection were obtained in the CP alignment by M2a, M3, and M8. Models M3 and M8 predicted similar parameters and showed 0.8% of the sites were under a very strong positive selection pressure (*ω* = 3.836 and 4.081, respectively) (Table 3). Bayesian methods assigned two sites 12S and 155R to the positively selected group with posterior probability >75% for 12S and >95% for 155R (Table 3).

## Discussion

The objectives of this investigation were to better understand the sequence diversity and genetic structure of BBSV population using different approaches. The four new Iranian BBSV isolates sequenced in this study (ac. no. MH705129 to MH705132) were all 3644 nts in length, with genome organization identical to that of the type BBSV member. The presented sequence data is the most common variant within each sample. Phylogenetic analysis using 3′UTR region grouped the BBSV isolates into two groups. Most of the BBSV isolates from China, Europe, and one from North America were in phylogroup GII. Group GI isolates from Iran fell into two subgroups. Almost all of the BBSV isolates from North-East Iran (Khorasan district, geographically the nearest county to Xinjiang province in China) were not clustered with Chinese or European isolates in Group II. The Iranian BBSV isolates in the subgroup II-IranC originating from North-West Iran (Kermanshah district, Fig 2) were clustered in Group II. Overall, the PATRISTIC plots using ML trees indicated that the coding and 3′UTR regions were closely linked and showed similar evolutionary pattern (Fig 3).

Different methods were used for recombination breakpoint prediction. Two recombination loci at different genomic locations were identified. According to the adopted criteria, a clear intra-recombination around nucleotide 668 in the RT-ORF1 and nucleotide 2714 in the ORF6 was found in Xinjiang/m81 isolate from subgroup II-Chinese (event 1). A putative recombination region around nucleotides 3521 to 3539 of 3′UTR region (Figure D in S2 File, Table C in S1 File) was detected among Iranian isolates in subgroup I-IranA. However, this recombination event was not supported with a high degree of confidence (Table 1). Evolutionary comparisons of a large number of isolates from mid-Eurasia, and East-Asia with representative worldwide isolates would be required to determine extent of recombination and genetic variability of BBSV.

Differentiation is considered one of the key subjects in population genetics. We have compared levels of diversification among BBSV subgroups. We used the *F*_*ST*_ program to measure the overall genetic variation between subpopulations. The range of *F*_*ST*_ is from zero (complete sharing of genetic sequences) to 1.0 (populations completely isolated from each other) [26]. The high *F*_*ST*_ values (> 0.6) were estimated among BBSV populations by DNASP [19] calculation of *F*_ST_. Pairwise comparisons between Group I and Group II isolates, of four genes and the 3′UTR are presented in Table D in S1 File. The overall values of *F*_*ST*_ for the RT-ORF1, ORF3, ORF4 and ORF6 were 0.818, 0.836, 0.822 and 0.805, respectively. For the majority of pairwise comparisons, *F*_ST_ values are supporting population differentiation for BBSV.

Haplotype and nucleotide diversity values were also compared to determine if BBSV population expansions have occurred. The mean nucleotide diversity of each gene and the within-group diversity were estimated to be similar to those reported for other plant viruses (Table 2) [11]. This finding indicates that BBSV populations are genetically stable. In addition, these analyses showed that, although the population sizes vary between BBSV groups, the rates of evolution of the ORFs analyzed were alike. The highest nucleotide diversity was found in Chinese isolates. However pairwise nucleotide identity, haplotype diversity, and nucleotide diversity revealed two subpopulations of closely related BBSV isolates in North-West and North-East of Iran (Table 2).

Nucleotide diversity estimates the average pairwise difference among sequences. Haplotype diversity is calculated based on the frequency and number of haplotypes in a sample. Estimates of nucleotide diversity can range from zero (no variation) to 0.1 (extreme divergence) between alleles, whereas haplotype diversity may differ between zero and 1.0 [27]. The haplotype and nucleotide diversity values for BBSV subpopulations are presented in Table 2. In most cases haplotype diversity values are high (from 0.666 to 1.0) and nucleotide diversity values are low (from 0.006 to 0.045). Generally, the combination of high haplotype diversity and overall absence of nucleotide diversity within individual subpopulations are consistent with a model of recent population expansion events. Given that evolutionary bottlenecks/founder effects [28], or strong selection pressures (e.g. due to host adaptation) would yield the same low genetic diversity. Independent statistical tests of population differentiation are necessary to better understand the evolutionary forces which influence the BBSV population.

Selection pressure is an important evolutionary force, which accelerates the variation between homologous proteins [29]. The *dNS*/*dS* ratio for coding regions (Table 2) was similar to other plant RNA viruses, indicating that they are under negative (purifying) selection [11]. The *dNS*/*dS* ratios differed for phylogenetic groups in RT-ORF1 and ORF6, indicating that the isolates in subgroup I-IranB are probably under positive selection. However, by this analysis, the I-IranA and II-Chinese subgroups are under negative selection (Table 2). The *dNS/dS* ratio showed that ORF3 was under purifying selection in both of these subgroups whereas ORF4 was under purifying and positive selections in I-IranA and II-Chinese subgroups, respectively (Table 2).

Most of the amino acid positions of functional proteins are considered to be conserved, while evolutionary fitness most possibly affects only a few sites [30]. The proteins encoded by the BBSV genome are all presumed to be essential to viral function and evolutionary constraints may well differ among them (Table 3). Coat proteins are multifunctional in plant viruses [31]. E.g. in *Tombusvirus*, CP is involved with nucleic acid binding and encapsidation [10]. Negative selection pressure was detected, in a few codons in coat proteins undergoing positive selection, indicating that variations in this gene can change viral fitness/infectivity [10, 32, 33].

BBSV has the highest sequence identity with *Tobacco necrosis virus*-TNV-D [34]. As previously reported for the CP of TNV-D [34], four conserved amino acids (117D, 120D, 179T, 232N) are involved in calcium binding. This four amino acid motif was also detected in BBSV-CP.

According to the comparison of CP amino acid sequences, Mehrvar [35] proposed that pathogenesis of some Iranian BBSV isolates on sugar beet correlates with changes at CP amino acid positions 12, 145 and 158. In our data, although CP gene sites are strongly conserved (*ω* = 0.041), only 0.8% of the sites were found to be under strong positive selection (*ω*_*2*_ = 3.838) (Tables 3). High *ω* ratios were detected for two of the amino acid residues (12S and 155R). Interestingly, 155R was conserved in all BBSV isolates whereas 12S was found only in Chinese isolates (Table 4).

**Table 4:** Amino acids sites putatively affected by positive selection in different ORFs products of *Beet black scorch virus*.

ORFs	Positively selected sites	BBSV subgroups	Function
RT-ORF1	15V	I-IranA,I-IranB&II-Chinese	82 kDa protein is required for RNA replication.
	39L	I-IranA&I-IranB	
	152T	I-IranA,I-IranB&II-Chinese	
	188Q	I-IranA	
	461T	I-IranA&II-Chinese	
	641S	I-IranA&II-Chinese	
	672A	I-IranA&II-Chinese	
	711I	I-IranA&I-IranB	
ORF4	8Q	II-Chinese	Cell to cell movement of the virus, accumulation of viral RNAs, induction of local lesions on *Ch*. *amaranticolor*.
ORF6	12S	II-Chinese	Four conserved amino acids involved in Ca^2+^ binding site as well as the plant virus icosahedral capsid protein “S” signature; CP phosphorylation plays an essential role in long-distance movement of BBSV that involves formation of stable virions.
	155R	I-IranA,I-IranB&II-Chinese	

Three overlapping ORFs (ORF3, ORF4 and ORF5) of BBSV are involved in cell-to-cell movement, accumulation of viral RNAs, and production of local lesions in *Ch*. *amaranticolor* [33]. Protein 7a (ORF3) is the only protein that does not show positive selection. Strong selective constraints on ORF3 can be attributed to its key role(s) in viral functions. In addition, the absence of positively selected sites in this gene suggests that host associated selection is probably not a main factor affecting BBSV evolution. For protein 7b (ORF4), site 8Q was found to be under positive selection only in II-Chinese subgroup (Table 4). Overall, this analysis indicates that purifying selection is acting to maintain functional integrity of BBSV proteins (Tables 3 and 4).

The low *ω* ratios determined for RT-ORF1 indicates that most of the amino acids were under purifying selection (Table 3), this was expected because of the role of this ORF in virus replication. Members of family *Tombusviridae* express their RdRp by translational readthrough strategy. This process is stimulated by an RNA structure that is positioned immediately downstream of the recoding site (readthrough stem-loop, RTSL), and a sequence in the 3′UTR (distal readthrough element, DRTE). A base pairing interaction between RTSL and DRTE is required for enhancement of readthrough. Any change in RNA sequences and structures that flanking either RTSL or DRTE may affect optimal translational readthrough and virus infectivity [36].

Differences in selection pressure on BBSV proteins may reflect diverse geographical origin of those proteins (later assembled into group-specific genomes by recombination); further selection pressure may arise after viral migration to different regions (Table 4). Chiba et al. [37] indicated that vigorous positive pressure on the *p25* gene of Italian BNYVV isolates facilitates their ability to overcome *Rz1*-host resistance genes, when other geographically bounded BNYVV strains could not.

Positive selection acting directly on amino acids with important roles is rarely illustrated because the adaptation-related phenotypic results of particular amino acids are generally unknown. Therefore, the precision of site-specific tests of selection yet remains basically in question. Nevertheless, if any sites are positively selected along a gene, it is possible that these sites are involved in increasing fitness. In addition, further study particularly using reverse genetic approaches is needed for a better understanding of the impact of the amino acid replacements. This is especially interesting in studying the extent to which extent positive selection can be attributed wholly to the efficacy of pathogen-host interactions, or if there are other forces resulting in positive selection on BBSV population.

This analysis is, to our knowledge, the first demonstration of the population structuring of BBSV in mid-Eurasian Iran. We have demonstrated effects of selection pressure and recombination in the evolution of BBSV. The phylogenetic relationships and comparisons between each virus group provide an understanding of evolutionary mechanisms. In addition, an understanding of the inter-specific diversification in the groups may be useful in developing strategies for controlling the diseases and the spread of BBSV. In this respect for future research projects on BBSV, we cloned CP gene and generated antibodies against bacterially expressed recombinant CP (rCP) of Iranian BBSV isolate. The polyclonal antibody was specific to BBSV and it was used successfully for BBSV detection in sugar beet samples.

## Conclusions

The evolutionary analysis of BBSV indicates that: a) Genetic differentiation has occurred among two original populations of BBSV (Table B in S1 File), one in the Middle East (Iran) and the other one in East Asia. b) Differentiation has divided Iranian BBSV isolates into two groups. This suggests the wide spread dissemination of the virus in sugar beet growing areas in Iran. c) Three BBSV subpopulations have been observed in Iran (based on the analysis of 3′UTR sequences). The subpopulation in North-East Iran appears to have diverged most recently. d) Recombination has a considerable role in the evolution of viruses by decreasing mutational bar, producing genetic diversification, and generating new strains. Our analysis using RDP4 [20] indicated a recombination event in the Chinese Xinjiang/m81 isolate and a putative inter-recombination breakpoint in 3′UTR of Iranian isolates in subgroup I-IranA. e) Fitness to the host plant or to the chytrid vector may illustrate how diversifying selection influences different sites in the BBSV genome. Furthermore, the positively selected site(s) in different ORFs of BBSV indicate(s) differentiation among evolved subgroups (Tables 3 and 4). BBSV isolates belonging to subgroups I-IranA and I-IranB are dispersed in Iran, whereas except for the USA isolate, all other isolates in BBSV subgroup II-Chinese were collected from China in East Asia. As previously described by Moury [38] different evolution patterns may have originated by biological variation and/or dispensation diversities. Beet has long been cultivated in Iran and some parts of Iran are considered as an origin point of the domestic beet [39]. However, improved beet seeds were introduced about 120 years ago to Iran. At that time each original population of BBSV might have passed through sequential bottleneck transmissions in different host varieties. These host changes may have selected for the introduction of changes at various sites during several generations. In addition, resting spores of *O*. *brassica* can remain dormant in infested soil for long times; e.g. as reported for *Polymyxa betae*, the vector of BNYVV [40]. Beet soil-borne viruses are transmitted primarily via the movement of soils containing viruliferous resting vector spores [40], so the transmission of BBSV from unknown natural hosts to sugar beet fields may well have been an important factor in this evolution.

## Supporting information

S1 File Occurrence of BBSV in soil samples (Table A). BBSV isolates analyzed in this study (Table B). Crossover sites in BBSV isolates detected using recombination detecting programs (Table C). Genetic differentiation analysis of BBSV isolates (Table D).
(DOCX)

S2 File ML trees and two dimensional of nucleotide diversity plot for RT-ORF1 (Figure A), ORFs (3+4) (Figure B), ORF6 (Figure C). 3′UTR recombination analysis (Figure D).
(DOCX)
