# Supplementary material for: Positive selection and recombination shaped the large genetic differentiation of Beet black scorch virus population
Source: PLoS One. 2019 Apr 25;14(4):e0215574. doi: 10.1371/journal.pone.0215574 (PMC6483173; doi:10.1371/journal.pone.0215574)
Supplement: S1 File — Occurrence of BBSV in soil samples (Table A). BBSV isolates analyzed in this study (Table B). Crossover sites in BBSV isolates detected using recombination detecting programs (Table C). Genetic differentiation analysis of BBSV isolates (Table D). (DOCX) [file pone.0215574.s001.docx]

S1 Table A Occurrence of *Beet black scorch virus* in soil samples collected from Khorasan Razavi

| Field Number | County | No. of root samples | |
| --- | --- | --- | --- |
|  |  | Tested | Infected ^a^ (%) |
| 1 | Jovein | 18 | 6 (33.33) |
| 2 | Jovein | 16 | 4 (25.00) |
| 3 | Jovein | 17 | 3 (17.64) |
| 4 | Jovein | 14 | 4 (28.57) |
| Total ^b^ |  | 65 | 17 (26.15) |
| 5 | Sarakhs | 19 | 0 (0.00) |
| 6 | Sarakhs | 16 | 2 (12.50) |
| 7 | Sarakhs | 19 | 4 (21.05) |
| Total ^b^ |  | 54 | 6 (11.11) |
| 8 | Taibad | 18 | 3 (16.66) |
| 9 | Taibad | 20 | 4 (20.00) |
| 10 | Taibad | 15 | 3 (20.00) |
| Total ^b^ |  | 53 | 10 (18.86) |
| Total ^b^ | | 172 | 33 (19.18) |

^a^ identification is based on serological reactions (ELISA).

^b^ average of virus infection.

S1 Table B *Beet black scorch virus* isolates analyzed in this study

| Isolate/variant | Location (city, district) | Ac. no. (genome region) |
| --- | --- | --- |
| **Asia** |  |  |
| -/- | China | AF452884 (Full) |
| Xinjiang/m294 | China/ Xinjiang/- | JN635330 (Full) |
| Xinjiang/m81 | China/ Xinjiang/- | JN635329 (Full) |
| Xinjiang/m163 | China/ Xinjiang/- | JN635328 (Full) |
| Xinjiang/m149 | China/ Xinjiang/- | JN635327 (Full) |
| Xinjiang/- | China/ Xinjiang/- | AY626780 (Full) |
| Ir-Ksh10/- | Iran/Kermanshah/Islam Abad Gharb | FN543422 (3′UTR) |
| Ir-Msh/- | Iran/Khorasan Razavi/Mashhad | FN543420 (3′UTR) |
| Ir-Ksh9/- | Iran/Kermanshah/- | FN543419 (3′UTR) |
| Ir-Ksh5/- | Iran/Kermanshah/Abbas Abad | FN543418 (3′UTR) |
| Ir-Ksh4/- | Iran/Kermanshah/Abbas Abad | FN543417 (3′UTR) |
| Ir-Ksh8/- | Iran/Kermanshah/Islam Abad Gharb | FN543416 (3′UTR) |
| Ir-Kh4/- | Iran/Khorasan Razavi/Golgei Rokh | FN543415 (3′UTR) |
| Ir-Ksh7/- | Iran/Kermanshah/Islam Abad Gharb | FN543414 (3′UTR) |
| Ir-Kh5/- | Iran/Khorasan Razavi/Fariman, Shahan Garmab | FN543413 (3′UTR) |
| Ir-Bj1/- | Iran/Khorasan Shomali/Boujnord | FN543412 (3′UTR) |
| Ir-Kh1/- | Iran/Khorasan Razavi/Chenaran | FN543411 (3′UTR) |
| Ir-Kh3/- | Iran/Khorasan Razavi/Torbat Jam | FN543410 (3′UTR) |
| Ir-Kh2/- | Iran/Khorasan Razavi/Chenaran | FN543409 (3′UTR) |
| Ir-Gh1/- | Iran/Ghazvin/Boein Zahra | FN543408 (3′UTR) |
| Ir-Kr1/- | Iran/Kerman/Bardsir, Dashtkar | FN543407 (3′UTR) |
| Ir-Ksh2/- | Iran/Kermanshah/Rostam Abad | FN543406 (3′UTR) |
| Ir-AzGh2/- | Iran/Azarbaeijan Gharbi/Bokan, Ghazmian | FN543405 (3′UTR) |
| Ir-Ha3/- | Iran/Hamadan/ Asad Abad, Mosi Abad | FN543404 (3′UTR) |
| Ir-Ha4/- | Iran/Hamadan/Nahavand, Gian | FN543403 (3′UTR) |
| Ir-AzGh3/- | Iran/Azarbaeijan Gharbi/Mohammad Yar | FN543402 (3′UTR) |
| Ir-Ksh1/- | Iran/Kermanshah/Dolah | FN543421 (Full) |
| Ir-Msh1/- | Iran/Mashhad/- | FN565520 (Full) |
| Ir-Sh1/- | Iran/Khorasan Shomali/Shirvan | FN543471 (Full) |
| Ir-Ha1/- | Iran/Hamadan/Malayer-Dehno Avard Zaman | FN543470 (Full) |
| Ir-AzGh1/- | Iran/Azarbaeijan Gharbi/Mohammad Yar | FN543469 (Full) |
| Ir-Ksh6/- | Iran/Kermanshah/- | FN543468 (Full) |
| Ir-Ksh3/- | Iran/Kermanshah/Islam Abad Gharb | FN543467 (Full) |
| Ir-Ha2/- | Iran/Hamadan/Asad Abad, Lak Lak | FN543466 (Full) |
| Val25/- | Iran/Khorasan Razavi/Torbat-Hydarieh | EU545828 (Full) |
| IRN.Kh29 | Iran/Khorasan/Jovain | MH705129 (Full) |
| IRN.Kh30 | Iran/Khorasan/Taibad | MH705130 (Full) |
| IRN.Kh32 | Iran/Khorasan/Sarakhs | MH705131 (Full) |
| IRN.Kh111 | Iran/Khorasan/Jovain | MH705132 (Full) |
| **Europe** |  |  |
| FR2/- | France/Yevre la Ville/- | AM941668 (3′UTR) |
| D1/- | Germany/-/- | AM941669 (3′UTR) |
| IT-CP/- | Italy/Copparo/- | AM941667 (3′UTR) |
| IT-C/- | Italy/Casale/- | AM941666 (3′UTR) |
| NL1/- | Netherlands/Monsma-Dronten/- | AM941670 (3′UTR) |
| Bu-Pm/- | Spain/Burgos/Pampliega | AM941671 (3′UTR) |
| CR-Dm2/- | Spain/Ciudad Real/Daimiel | AM941663 (3′UTR) |
| CR-Dm1/- | Spain/Ciudad Real/Daimiel | AM941662 (3′UTR) |
| Va-Be/- | Spain/Valladolid/Bercero | AM941661 (3′UTR) |
| Le-Pb/- | Spain/Leon/Pobladura | AM941660 (3′UTR) |
| Le-LN/- | Spain/Leon/Laguna de Negrillos | AM941659 (3′UTR) |
| Ab-My/- | Spain/Albacete/Minaya | AM941658 (3′UTR) |
| GB-K/- | United Kingdom/Knodishall/- | AM941665 (3′UTR) |
| GB-B/- | United Kingdom/Blaxhall/- | AM941664 (3′UTR) |
| **North America** |  |  |
| Co/- | USA/Colorado/- | EF153268 (Full) |

S1 Table C Crossover sites in *Beet black scorch virus* isolates detected using recombination detecting programs

| Event (2) | Recombinant  (Ac. No) | Parental isolates (Ac. No.) ^a^ | |  | Breakpoints ^b^ | |  | Methods | | | | | |
| --- | --- | --- | --- | --- | --- | --- | --- | --- | --- | --- | --- | --- | --- |
|  |  | Major parent | Minor parent |  | Begin | End |  | RDP | GENECONV | BootScan | Maxchi | Chimaera | Siscan |
| 1 | Ir-Msh (FN543420), Ir-Kh1 (FN543411), Ir-Ha3 (FN543404), Ir-Msh1 (FN565520), Ir-Kh2 (FN543409), Ir-Kh2 (FN543470) | Ir-Ksh9  (FN543419) | Ir-Ksh5  (FN543418) |  | ND^d^ | 3521/3*′* UTR |  | ND | 6.328×10^-1^ | ND | 6.937×10^-2^ | ND | **1.210×10^-5^** |
| 7 | Ir-Kh5  (FN543413) | Ir-Ksh9  (FN543419) | Ir-Ksh5  (FN543418) |  | ND | 3527/3*′* UTR |  | ND | ND | ND | 1.206×10^-1^ | ND | ND |
| 8 | Ir-Kh4  (FN543415) | Ir-Ksh9  (FN543419) | Ir-Ksh5  (FN543418) |  | ND | 3527/3*′* UTR |  | 0.448 | ND | ND | **6.198×10^-3^** | ND | 1.979×10^-1^ |
| 9 | Ir-Kh3  (FN543410) | Ir-Ksh9  (FN543419) | Ir-Ksh5  (FN543418) |  | ND | 3532/3*′* UTR |  | 0.344 | ND | ND | ND | ND | **3.416×10^-3^** |
| 10 | Val25  (EU545828) | Ir-Ksh9  (FN543419) | Ir-Ksh5  (FN543418) |  | ND | 3527/3*′* UTR |  | **3.662×10^-3^** | 1.215×10^-1^ | ND | 3.415×10^-3^ | ND | 7.587×10^-2^ |
| 11 | Ir-Sh1  (FN543471) | Ir-Ksh9  (FN543419) | Ir-Ksh5  (FN543418) |  | ND | 3534/3*′* UTR |  | ND | 3.847×10^-1^ | ND | 1.074×10^-1^ | ND | **8.868×10^-4^** |
| 12 | Ir-Bj1  (FN543412) | Ir-Ksh9  (FN543419) | Ir-Ksh5  (FN543418) |  | ND | 3527/3*′* UTR |  | 0.181 | 2.508×10^-1^ | ND | **3.507×10^-3^** | ND | 1.979×10^-1^ |
| 13 | Ir-Gh1  (FN543408) | Ir-Ksh9  (FN543419) | Ir-Ksh5  (FN543418) |  | ND | 3539/3*′* UTR |  | 0.104 | 1.540×10^-1^ | 6.323×10^-2^ | 5.734×10^-2^ | 1.031×10^-2^ | **3.535×10^-6^** |
| 14 | Ir-Kr1  (FN543407) | Ir-Ksh9  (FN543419) | Ir-Ksh5  (FN543418) |  | ND | 3539/3*′* UTR |  | ND | 1.820×10^-1^ | 7.896×10^-2^ | 2.974×10^-2^ | ND | **9.313×10^-7^** |
| 15 | Ir-Ksh2  (FN543406) | Ir-Ksh9  (FN543419) | Ir-Ksh5  (FN543418) |  | ND | 3534/3*′* UTR |  | ND | ND | 1.577×10^-1^ | 1.046×10^-1^ | ND | **6.923×10^-5^** |
| 16 | Ir-AzGh2  (FN543405) | Ir-Ksh9  (FN543419) | Ir-Ksh5  (FN543418) |  | ND | 3534/3*′* UTR |  | 0.241 | 3.225×10^-1^ | 7.680×10^-1^ | 3.209×10^-3^ | ND | **6.923×10^-5^** |
| 17 | Ir-AzGh3  (FN543402) | Ir-Ksh9  (FN543419) | Ir-Ksh5  (FN543418) |  | ND | 3534/3*′* UTR |  | ND | 1.904×10^-1^ | 9.577×10^-1^ | 4.663×10^-3^ | ND | **1.608×10^-5^** |
| 18 | Ir-Ksh1  (FN543421) | Ir-Ksh9  (FN543419) | Ir-Ksh5  (FN543418) |  | ND | 3527/3*′* UTR |  | 2.014×10^-2^ | 1.215×10^-1^ | ND | **3.415×10^-3^** | ND | 7.587×10^-2^ |
| 19 | Ir-Ha4  (FN543403) | Ir-Ksh9  (FN543419) | Ir-Ksh5  (FN543418) |  | ND | 3527/3*′* UTR |  | 2.014×10^-2^ | 1.215×10^-1^ | ND | **3.415×10^-3^** | ND | 7.587×10^-2^ |
| 20 | Ir-AzGh1  (FN543469) | Ir-Ksh9  (FN543419) | Ir-Ksh5  (FN543418) |  | ND | 3527/3*′* UTR |  | 2.014×10^-2^ | 1.215×10^-1^ | ND | **3.415×10^-3^** | ND | 7.587×10^-2^ |
| 21 | IRN.Kh29  (MH705129) | Ir-Ksh9  (FN543419) | Ir-Ksh5  (FN543418) |  | ND | 3534/3*′* UTR |  | ND | ND | ND | 3.784×10^-1^ | ND | **1.477×10^-1^** |

^a^ Parental isolate means the most likely isolate among analyzed isolates.

^b^ Numbers indicate recombination sites.

^c^ Recombination break points were analyzed using RDP, GENCONV, BOOTSCAN, MAXCHI, CHIMAERA and SISCAN methods, in the RDP4 v.4.70 [20] with the presented highest *P*-value being that determined by the method indicated in bold type.

^d^.Not determined

S1 Table D Analysis of genetic differentiation between geographical isolates populations of *Beet black scorch virus*

| ORFs | Comparison between subpopulations | *F_ST_* |
| --- | --- | --- |
| 3*′*UTR | I-IranA vs. I-IranB | 0.757 |
|  | I-IranA vs. II-IranC | 0.864 |
|  | I-IranA vs. II-Chinese | 0.859 |
|  | I-IranA vs. II-Europe | 0.899 |
|  | I-IranB vs. II-IranC | 0.850 |
|  | I-IranB vs. II-Chinese | 0.783 |
|  | I-IranB vs. II-Europe | 0.835 |
|  | II-IranC vs. II-Chinese | 0.837 |
|  | II-IranC vs. II-Europe | 0.892 |
|  | II-Chinese vs. II-Europe | 0.604 |
| RT-ORF1 | I-IranA vs. I-IranB | 0.897 |
|  | I-IranA vs. II-IranC | 0.770 |
|  | I-IranB vs. II-Chinese | 0.841 |
| ORF3 | I-IranA vs. I-IranB | 0.828 |
|  | I-IranA vs. II-IranC | 0.802 |
|  | I-IranB vs. II-Chinese | 0.837 |
| ORF4 | I-IranA vs. I-IranB | 0.855 |
|  | I-IranA vs. II-IranC | 0.834 |
|  | I-IranB vs. II-Chinese | 0.726 |
| ORF6 | I-IranA vs. I-IranB | 0.899 |
|  | I-IranA vs. II-IranC | 0.874 |
|  | I-IranB vs. II-Chinese | 0.861 |
