# Supplementary material for: Positive selection and recombination shaped the large genetic differentiation of Beet black scorch virus population
Source: PLoS One. 2019 Apr 25;14(4):e0215574. doi: 10.1371/journal.pone.0215574 (PMC6483173; doi:10.1371/journal.pone.0215574)
Supplement: S2 File — ML trees and two dimensional of nucleotide diversity plot for RT-ORF1 (Figure A), ORFs (3+4) (Figure B), ORF6 (Figure C). 3′UTR recombination analysis (Figure D). (DOCX) [file pone.0215574.s002.docx]

Maximum likelihood (ML) tree using 20 RT-ORF1 sequences indicated two main groups. Group I included two subgroups. All Iranian isolates fell into I-IranA and Chinese isolates with the Co isolate from USA (Accession No. EF153268) fell into Chinese subgroup (Figure A). Three Iranian isolates from North West (Ir-Ha2, Ir-Ksh3, and Ir-Ksh6) diverged from other Iranian isolates and fell into group II. Two-dimensional pairwise nucleotide distances plot analysis also revealed two main groups representation of two phylogenetic groups. BBSV isolates in subgroup I-IranA showed the lowest nucleotide diversity (0.000 to 0.044; highest similarities), whereas nucleotide diversities for subgroup I-IranB was 0.111 to 0.129. The Co isolate was distinct from Chinese isolates in Chinese subgroup with pairwise nucleotide diversities ranging 0.044 to 0.111. The pairwise nucleotide distance 0.089 to 0.111 was indicated for Chinese subgroup (Figure A).


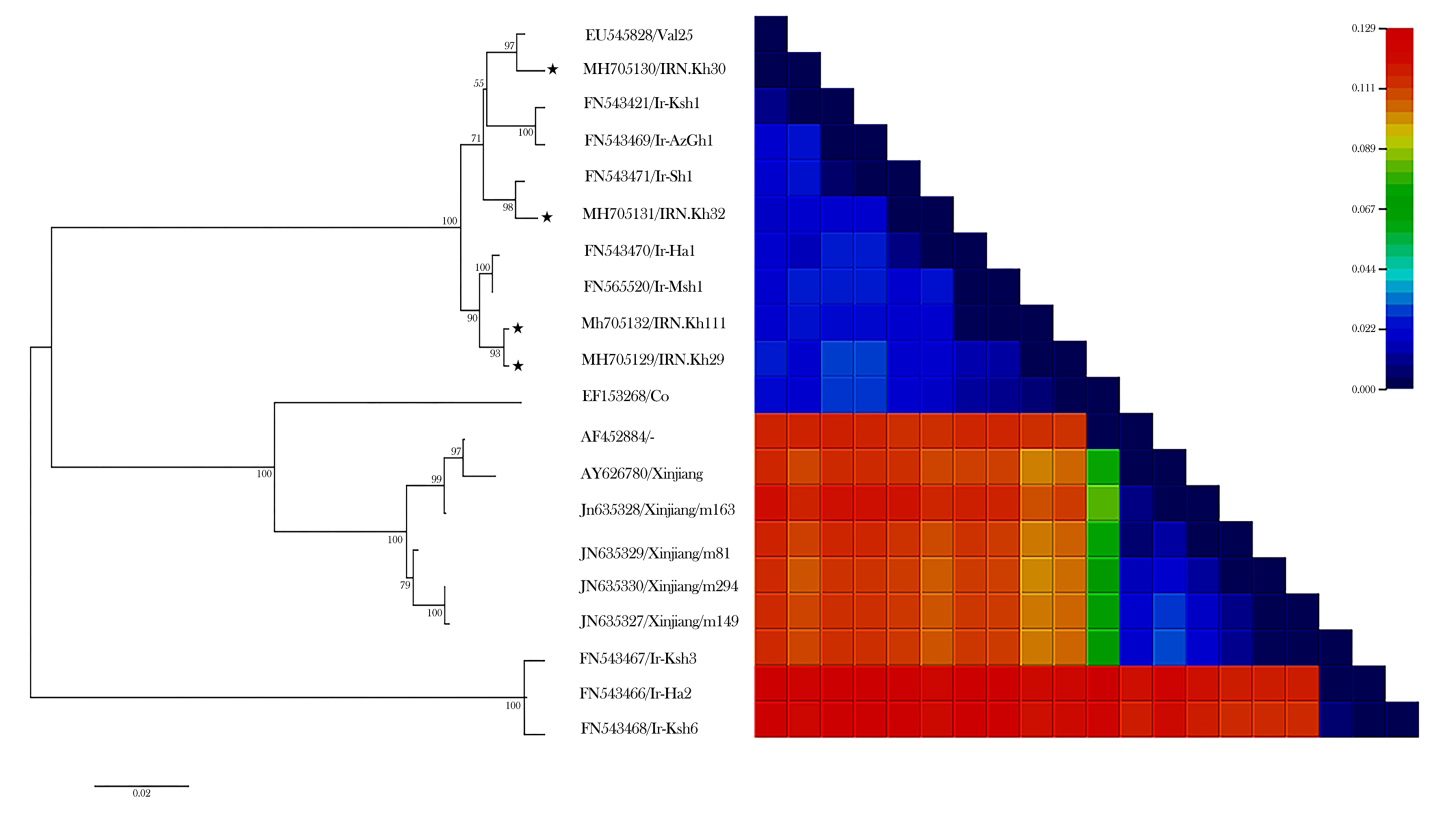


Figure A. Maximum likelihood (ML) tree and two dimensional nucleotide diversity plot showing the relationship among 20 RT-ORF1 sequences of *Beet black scorch virus* isolates. The tree was constructed using Kimura’s two-parameter (K2) model of nucleotide substitution with rate variation among sites modeled using a gamma distribution and a proportion of invariable sites (K2+G+I). Numbers at each node indicate the percentage of supporting puzzling steps (or bootstrap samples) in ML method. The name of each isolate and the country of its origin are listed in the accession number in the International Gene Sequence Database (GenBank). Iranian isolates sequenced in this study are indicated by star mark.

Maximum likelihood (ML) tree of concatenate ORFs (3+4) sequences also, showed two main groups GI and GII, with three subgroups. The three BBSV isolates from North-West (I-IranB) were clustered in a distinct subgroup in Group II, but I-IranA isolates fell into group I, which also was confirmed using two-dimensional pairwise nucleotide distances plot analysis (Figure B). The ORFs (3+4) sequences of Iranian BBSV isolates in GI showed the lowest nucleotide diversity (0.000 to 0.034; highest similarities). However high pairwise nucleotide distances (0.034 to 0.100) was indicated in phylogenetic GII, with pairwise nucleotide distances 0.069 to 0.086 and 0.086 to 0.100 for I-IranB and Chinese isolates, respectively (Figure B).


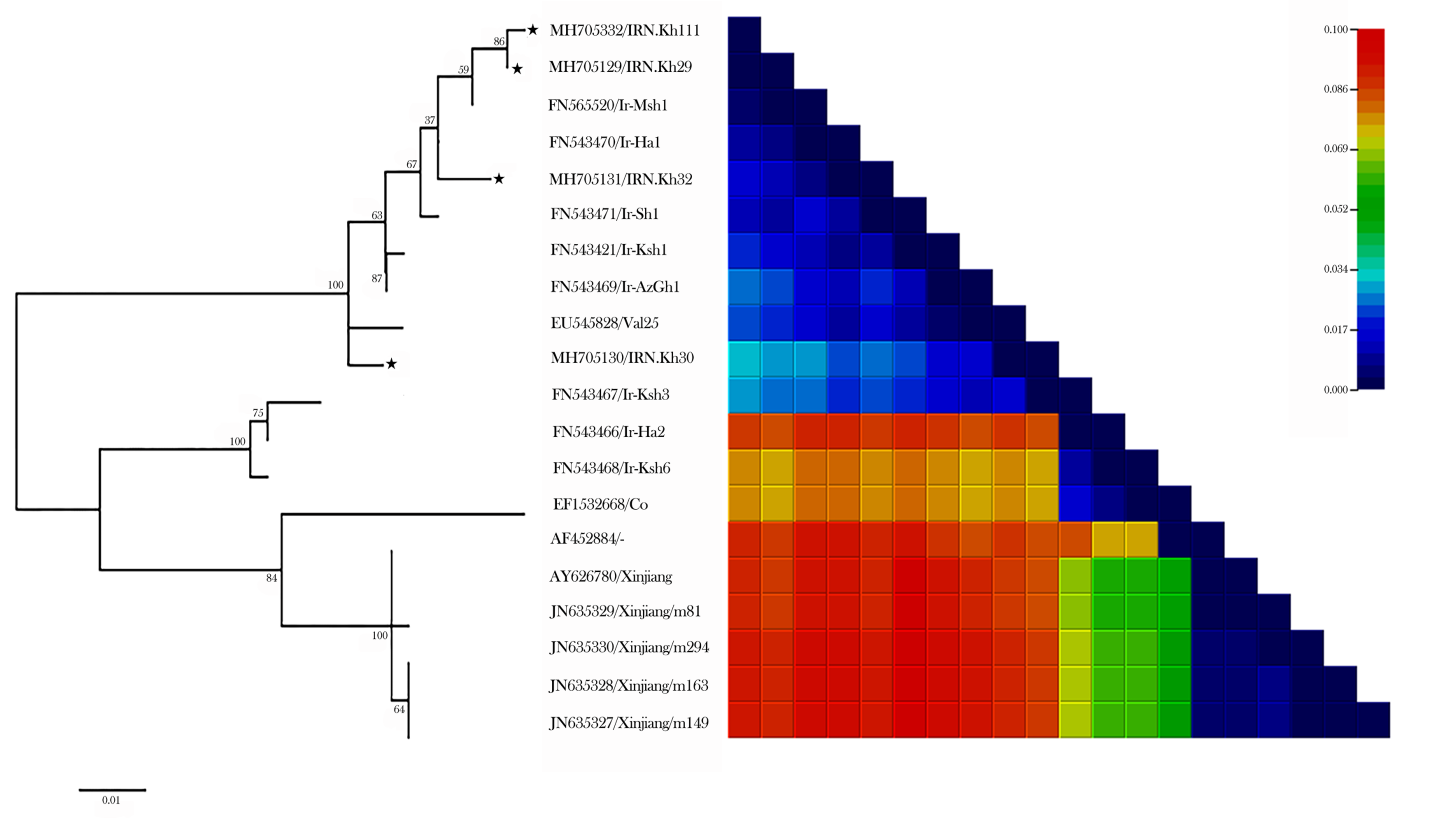


Figure B. Maximum likelihood (ML) tree and two dimensional nucleotide diversity plot showing the relationship among 20 ORFs 3+4 sequences of *Beet black scorch virus* isolates. The tree was constructed using Kimura’s two-parameter (K2) model of nucleotide substitution with rate variation among sites modeled using a proportion of invariable sites (K2+I). Numbers at each node indicate the percentage of supporting puzzling steps (or bootstrap samples) in ML method. The name of each isolate and the country of its origin are listed in the accession number in the GenBank. Iranian isolates sequenced in this study were indicated by star mark.

The topology of the ML tree constructed using the CP sequences of BBSV, was similar to that for RT-ORF1 gene, wherein the BBSV isolates fell in two main Groups (Figure C) Almost all of the Iranian isolates fell in I-IranA and I-IranB subgroups whereas, the Chinese isolates clustered in group II. Based on pairwise nucleotide diversities analysis the lowest nucleotide diversity (0.000 to 0.034; highest similarities) was detected in I-IranA subgroup. In addition, In Chinese subgroup, the USA isolate was grouped in a distinct branch with nucleotide diversities from 0.102 to 0.171. The highest pairwise nucleotide distance values ranging from 0.171 to 0.198 were indicated for I-IranB subgroup (Figure C).


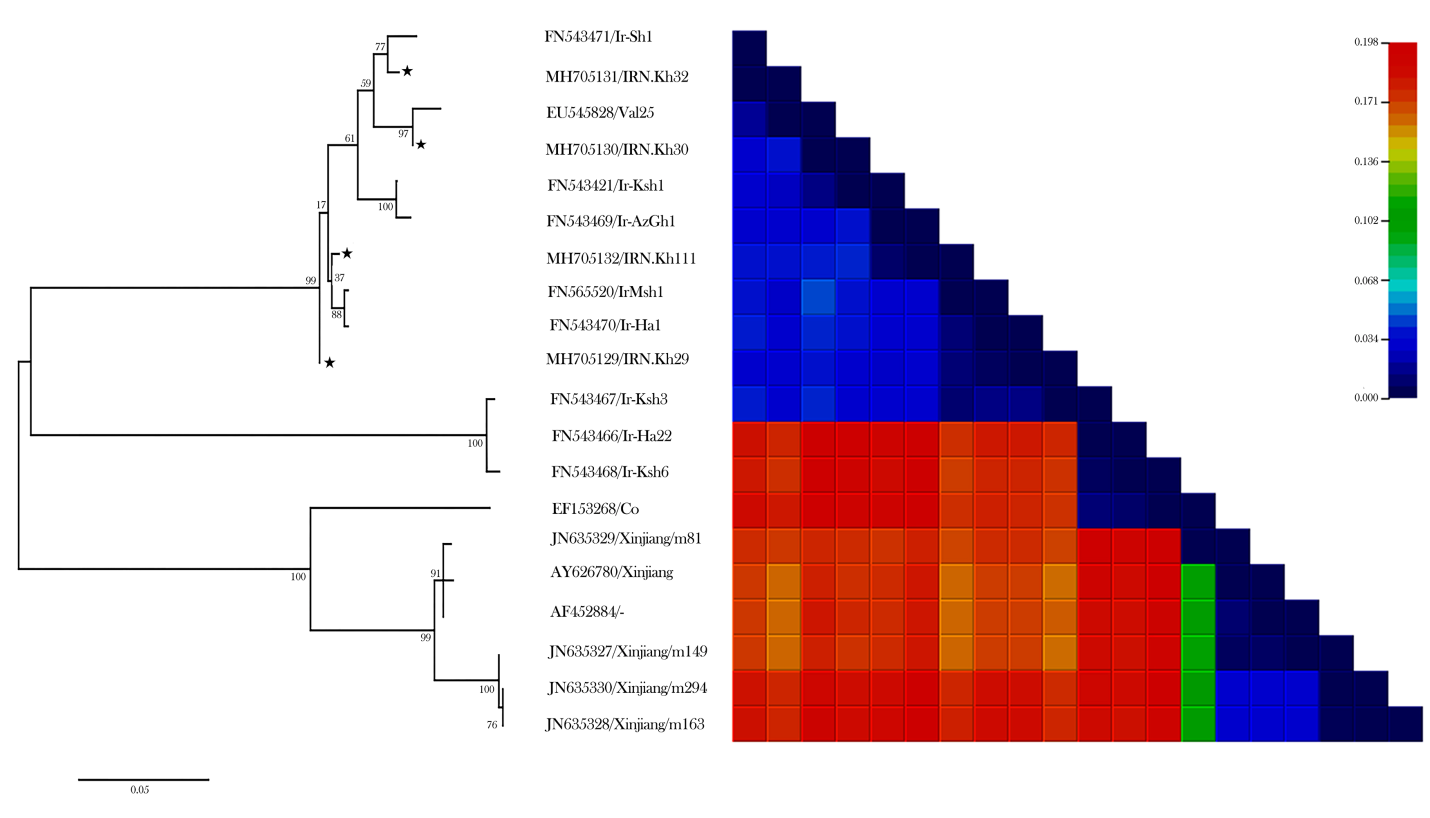


Figure C. Maximum likelihood (ML) tree and two dimensional nucleotide diversity plot showing the relationship among 20 ORF6 sequences of *Beet black scorch virus* isolates. The tree was constructed using Kimura’s two-parameter (K2) model of nucleotide substitution with rate variation among sites modeled using a gamma distribution (K2+G). Numbers at each node indicate the percentage of supporting puzzling steps (or bootstrap samples) in ML method. The name of each isolate and the country of its origin are listed in the accession number in the GenBank. Iranian isolates sequenced in this study were indicated by star mark.

Different methods were used for recombination breakpoint prediction and provided evidence for inter recombination event across 3*′*UTR region. A putative inter-recombination breakpoint (event 2) was detected using RDP4, in 3′UTR region of Iranian isolates in subgroup I-IranA (Figure D in S2 File, Table C in S1 File) with the likely parental isolates Ir-Ksh9 (FN543419) belonged to subgroup II-IranC and Ir-Ksh5 (FN543418) from subgroup I-IranB, as major and minor parents, respectively. However, the recombination event 2 did not support with a high degree of confidence (with multiple different methods and with a low associated *P*-value for each of the methods).


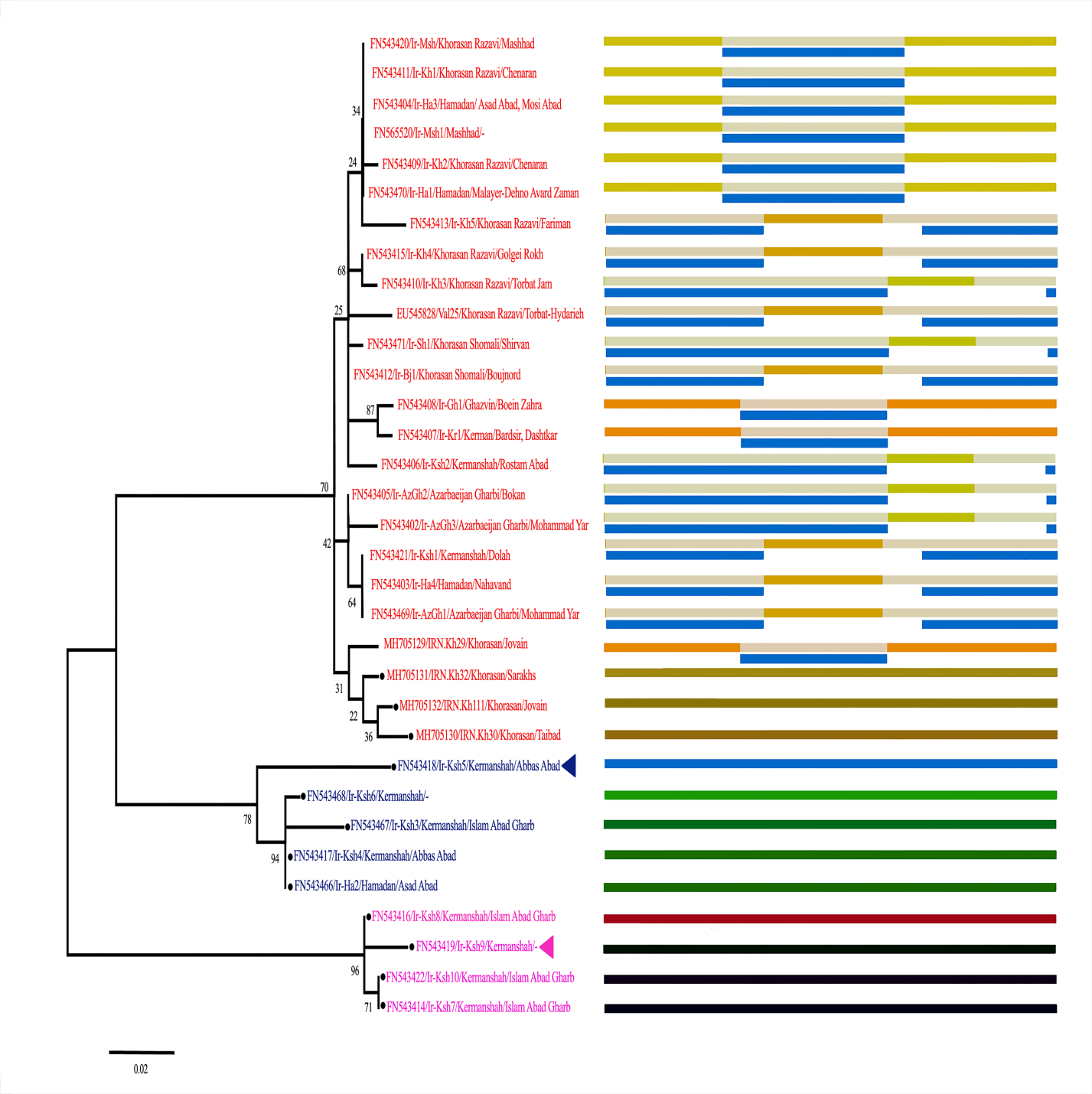


**Figure D. 3*′*UTR** r**ecombination analysis.**

Maximum likelihood (ML) tree and schematic putative recombinant isolates (event 2) which detected using the programs of the RDP4 software, showing for 3′UTR region of Iranian *Beet black scorch virus* isolates. The tree is constructed using (K2+G) model. Subgroups were highlighted by red, blue, and pink colors for IR-IA, IR-IB and IR-IIC, respectively. Non-recombinant isolates are indicated by black circle in the tree. Major (Ir-Kh9) and minor (Ir-Kh5) parents highlighted by pink and blue triangular in ML tree, respectively.
